# Limited life‐history plasticity in marginal population of an invasive foundation species: Unraveling the genetic underpinnings and ecological implications

**DOI:** 10.1002/ece3.11549

**Published:** 2024-06-08

**Authors:** Xincong Chen, Jiayu Wang, Wenwen Liu, Yihui Zhang

**Affiliations:** ^1^ Key Laboratory of the Ministry of Education for Coastal and Wetland Ecosystems College of the Environment and Ecology, Xiamen University Xiamen Fujian China

**Keywords:** biological invasion, genetic divergence, marginal population, phenotypic plasticity, reproductive phase, *Spartina alterniflora*

## Abstract

Plant's life history can evolve in response to variation in climate spatio‐temporally, but numerous multiple‐species studies overlook species‐specific (especially a foundation species) ecological effects and genetic underpinnings. For a species to successfully invade a region, likely to become a foundation species, life‐history variation of invasive plants exerts considerable ecological and evolutionary impacts on invaded ecosystems. We examined how an invasive foundation plant, *Spartina alterniflora*, varied in its life history along latitudinal gradient using a common gardens experiment. Two common gardens were located at range boundary in tropical zone and main distribution area of *S. alterniflora* in temperate zone in China. Within each population/garden, we measured the onset time of three successive phenological stages constituting the reproductive phase and a fitness trait. In the low‐latitude garden with higher temperature, we found that reproductive phase was advanced and its length prolonged compared to the high‐latitude garden. This could possibly due to lower plasticity of maturity time. Additionally, plasticity in the length of the reproductive phase positively related with fitness in the low‐latitude garden. Marginal population from tropic had the lowest plasticity and fitness, and the poor capacity to cope with changing environment may result in reduction of this population. These results reflected genetic divergence in life history of *S. alterniflora* in China. Our study provided a novel view to test the center–periphery hypothesis by integration across a plant's life history and highlighted the significance in considering evolution. Such insights can help us to understand long‐term ecological consequences of life‐history variation, with implications for plant fitness, species interaction, and ecosystem functions under climate change.

## Graphical abstract

The ecological effects of the variation in the plant life history of specific species (e.g., foundation species) and genetic underpinnings therein were rarely studied. We found that geographic populations of invasive *Spartina alterniflora* had different plasticity in the reproductive phase in response to warming, and the lowest plasticity and fitness of southernmost population may suggest the slowdown of rapid dispersion of *S. alterniflora* in China. Our study provided a novel view to test the center–periphery hypothesis by integration across a plant's life history.

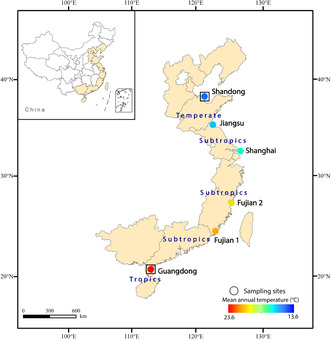

## 1 INTRODUCTION

The life history of an organism, from its birth through its growth and reproductive stages and ultimately to its death, all contributes to its fitness (Li et al., 2016; Stearns, 1992). For plants, the reproductive phase plays a critical role and is highly sensitive to variation in the environment (Caradonna & Bain, 2016; Iler et al., 2019). Furthermore, a certain life‐history stages may affect later ones (Donohue, 2002; Sola & Ehrlen, 2007), such that variation in environmental conditions could cause linked changes in multiple life‐history stages (Primack, 1987; Stearns, 2000). While a great deal of studies only focused on individual stages, integration across a plant's life history is necessary to evaluate how plant fitness responses to changing environment (Augspurger & Zaya, 2020; Li et al., 2016).

As the mean global temperature continues to increase (Masson‐Delmotte et al., 2021), organisms will have to adjust multiple stages of their life histories, often with different levels of plasticity and asynchronous response (Williams, 2018). As a result, some life‐history stages may respond more readily to changing environments than others. For example, in the face of increasing temperature, the reproductive phases of several alpine plants are more variable and have greater temperature sensitivity than other stages (Li et al., 2016). In addition to altering the timing in which changing environmental conditions influence plant life histories, these conditions can also change the interval between stages. Therefore, incorporating both timing and duration of life‐history stages is critical for a comprehensive understanding of how plant life history and fitness respond to environmental changes (Haggerty & Galloway, 2011; Post et al., 2008). Major studies on plant life history complied data from numerous species; however, they often presented mixed results (Collins et al., 2021; Li et al., 2016; Post et al., 2008; Sherry et al., 2007). Such condition could be due to that different species or taxa complicate the response to climate changes. Thus, studies that compiled a wealth of species data (Augspurger & Zaya, 2020; Guo et al., 2023) could not implicate the ecological consequences of life history in a specific species. Especially when the species is a foundation species, due to whose dominate role in providing the structure upon which ecosystems are built, the life‐history variation in a foundation species can have far‐reaching impact on the whole ecological communities. Additionally, the life‐history differences among populations of a specific species suggest the genetic underpinnings (Haggerty & Galloway, 2011), which could advance understanding of long‐term impacts of climate change on plant life history (Govaert et al., 2021). Nevertheless, such differences among populations were less discussed.

Biological invasions of species from outside of their native range can often interact with climate change (Wolkovich & Cleland, 2014). For a species to successfully invade a region, likely to become a foundation species, it is often necessary for it to have high plasticity in life history to changing environments (Gillman et al., 2015; Hulme, 2007; Maron et al., 2004). Furthermore, the ability of invasive plants to rapidly respond to changing environments via phenotypic plasticity can vary greatly among different populations (Maron et al., 2004). The populations located at the edges of species' ranges are of paramount importance for comprehending the future shifts in species distributions. It is in these regions where colonization and extinctions will predominantly occur under climate change (Mägi et al., 2011). As the center–periphery hypothesis stated that center and margin populations differ significantly in life‐history traits (e.g., demography, reproduction, and morphology) and plasticity, which can influence their acclimation under changing environments (Abeli et al., 2014; Latron et al., 2023). Generally, the fitness and performance of marginal populations are lower than center ones (Kawecki, 2008; Valladares et al., 2014). Despite the significance of integration across life‐history stages in fitness, the differences in integrated life history among populations were seldom studied.

Here, we investigated the life‐history responses of *Spartina alterniflora*, a widespread invasive salt marsh species, to latitudinal climate variation in China. While *S. alterniflora* is native to the eastern coast of the United States, it has become an aggressive invader around the world (Strong & Ayres, 2013). It was initially introduced into China in 1979 (Xu & Zhuo, 1985) at Luoyuan Bay, Fujian (mid‐latitude), and then distributed through anthropogenic introduction and natural dispersion to low and high latitudes (Chen et al., 2023; Qiao et al., 2019). This species is now a foundation species across the coast of China, distributing from the temperate to tropical zones (An et al., 2007; Zhang et al., 2017). In recent three decades, it has spread mainly to the temperate and subtropical zones (Zhang et al., 2023). There is considerable variation in the life history and reproduction among *S. alterniflora* populations in both their native (Seneca, 1974) and invasive (Liu & Zhang, 2021) ranges. Especially, the flowering time of the marginal populations from tropical zone was notably earlier than the temperate and subtropical ones in China (Chen et al., 2021). However, how this variation contributes to the reproductive phase is critical for understanding the adaptation of *S. alterniflora* in invasive China. To explore this, we established two common gardens, one in the southernmost latitude boundary (tropical zone) of *S. alterniflora*'s distribution in China and one in the main distribution area in the temperate zone. Such design allowed us to estimate the life‐history variation among *S. alterniflora* populations in experiencing warming. In each common garden, we measured three successive stages within reproduction phase of several widely distributed *S. alterniflora* populations. Our aim was to address the following questions: (1) How does the reproductive phase of different *S. alterniflora* populations vary between common gardens? (2) Whether the three stages constituting the reproductive phase have different degrees of variation (plasticity) between common gardens? (3) Dose this variation correlate with the fitness of populations along the latitudinal gradient? We hypothesize that (1) from the high latitude garden to the low latitude one, the rising temperature will advance and prolong the reproductive phase due to the enhanced physiological metabolism of plants; (2) the three stages constituting the reproductive phase have different degrees of variation between gardens due to their different plastic response to warming; and (3) the variation in the reproductive phase of *S. alterniflora* populations correlates with their fitness due to the effect of reproductive phase on plant fitness. By comparing the plasticity of integrated life‐history stages between marginal population with other ones and linking to the fitness, we reveal the genetic underpinnings in life‐history variation of *S. alterniflora* and the ecological implications of such variation.

## 2 MATERIALS AND METHODS

### 2.1 *Spartina alterniflora* populations sampling in field

**FIGURE 1:**
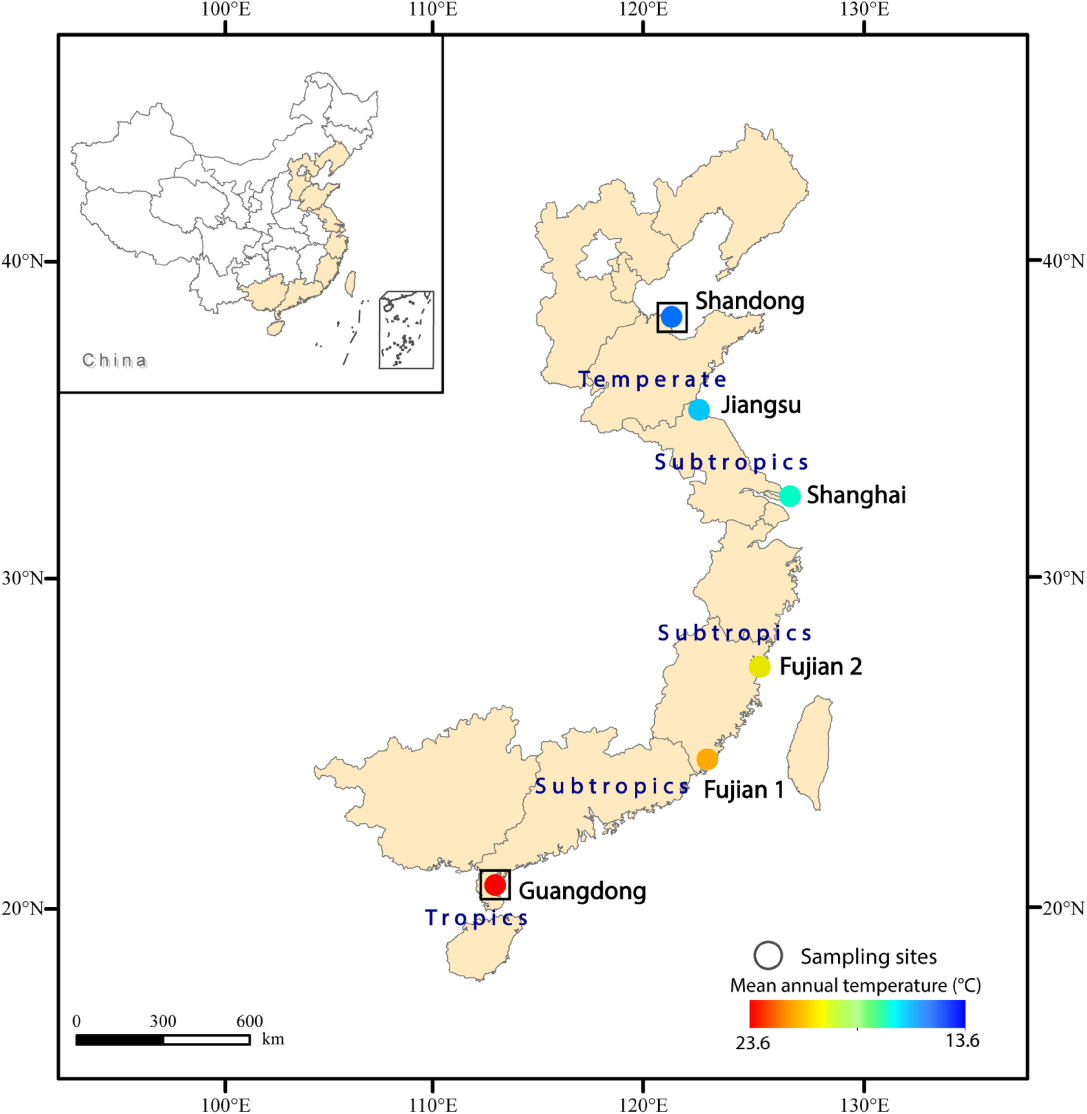
Map of study sites. Solid circles indicate locations of population collections. The color of the circles indicates the mean annual temperature of populations, and squares surrounding circles indicate common garden locations. Sites were Guangdong (21° N), Fujian 1 (24° N), Fujian 2 (26° N), Shanghai (32° N), Jiangsu (35° N), and Shandong (38° N).

At the end of growing season in 2018, we sampled six *S. alterniflora* populations across its latitudinal distribution (21° N to 38° N) in China, encompassing temperate, subtropical, and tropical climates, including a marginal population from tropic (Figure 1, Appendix S1: Table S1). At each site, we sampled low marshes where *S. alterniflora* dominated, whereas higher marshes tend to be dominated by mangroves or *Phragmites australis* in China (Li et al., 2009). We chose two subsites (2–3 km apart) per site and set five 0.5 × 0.5 m quadrats per subsite. Quadrats were at least 30 m apart and came from different *S. alterniflora* clones, and each quadrat was designated as a seed family. In each quadrat, we collected three mature intact inflorescences and picked out filled seeds which we stored separately in sealed plastic bags with 10 PSU seawater at 4°C.

### 2.2 Common garden experiment setup and sampling

To assess the variation in the life history of *S. alterniflora* under varying environmental conditions, we established two greenhouse common gardens: one near the southernmost boundary of its distribution (Guangdong, 21° N, tropical zone) and another within its main distribution area in temperate zone (Shandong, 38° N) (Figure 1, Appendix S1: Table S1). In Mar‐2019, we germinated seeds from each site and cultivated them in the greenhouse at Xiamen University, Fujian (24° N), until seedlings were 7–8 cm tall, at which point we transplanted them into each garden. We established 10 separate rectangular plastic pools (length: 1.1 m, width: 0.8 m, depth: 0.3 m) in each common garden and placed six plastic pots (18 cm diameter, 24 cm tall) in each pool. We filled pots with a mixture of 50% Jiffy's peat substrate (Jiffy Products International BV, Moerdijk, Netherlands) and 50% vermiculite by volume; this uniform culture reduced potential edaphic effects from the field. We then transferred one robust seedling from each quadrat of the six populations to a randomly assigned plastic pot in each pool (1 seedling × 10 quadrats × 6 populations × 2 gardens = 120 seedlings). We filled pools with 10 PSU seawater mixed from sea salt so that the water level was parallel to the soil level in the pots, supplied freshwater to the pools each day to maintain salinity, and changed water monthly. Although this method did not completely mimic the edaphic and tidal conditions that plants experienced in nature, the consistent treatments allowed us to make clear comparisons between plants from different locations.

After transplanting, we recorded the onset date of three successive stages that affect resource allocation between vegetative and reproductive tissues that have important consequences for population fitness: (i) flag leaf, which is the uppermost leaf of the plants expanded, is often considered to be the primary contributor to sexual reproduction in gramineous plants (Guo, Li, Zhou, & Yang, 2020; Li et al., 1998); (ii) flowering, when the inflorescence emerged from the uppermost leaf blade and bore visible pollen (Chen et al., 2021; Qiu et al., 2018); and (iii) mature, where the spikelets in the inflorescence of individual plant became yellow and not disperse yet, indicating the end of the reproductive phase (Guo, Li, & Yang, 2020; León‐Osper et al., 2020). We identified these stages every 4 days until the end of growing season (Nov‐2019). In each pot, we marked the first *S. alterniflora* shoot that began to have flag leaf, so that individual plants could be followed throughout the growing season. Previous study demonstrated *S. alterniflora* scaled their fecundity according to shoot sizes that larger shoots had greater fecundity (Liu & Pennings, 2019). We also observed in this study that the first shoot was almost always the highest and the most fecund one. Thus, the first shoot can reflect the resource allocation between vegetative and reproductive phase of the plant in a pot. Because inflorescence biomass is an appropriate proxy for plant fitness (Hakes & Cronin, 2011; Johnson et al., 2010), we harvested the intact inflorescences of the marked shoot when recorded the onset date of mature (seeds in the spikelets were also mature) in about August in the low latitude garden and October in the high latitude one. The inflorescences were dried at 60°C to constant mass and weighed them.

### 2.3 Climate variables

For each sampled population, we obtained mean annual temperature (MAT), mean coldest daily temperature (MCDT), mean warmest daily temperature (MWDT), and the annual range of temperature (calculated as MWDT – MCDT; TAR) (Alexander et al., 2012) from 1981 to 2019 from the China Meteorological Data Service Center (CMDC, http://data.cma.cn). This time period represents a large proportion of the time since *S. alterniflora* was introduced and following its introduction in 1979. In each common garden, we set a HOBO temperature logger (MX2202) 1 m above ground at the center in each garden to record temperature at 10‐minute intervals throughout the growing season. These temperature data were used to calculate climatic variables above, except the TAR. The mean annual temperature (MAT) and mean coldest daily temperature (MCDT) decreased with increasing latitude for each population and between the low and high latitude gardens. In addition, the annual range of temperature (TAR) increased with increasing latitude (Appendix S1: Table S1).

### 2.4 Data analyses

**FIGURE 2:**
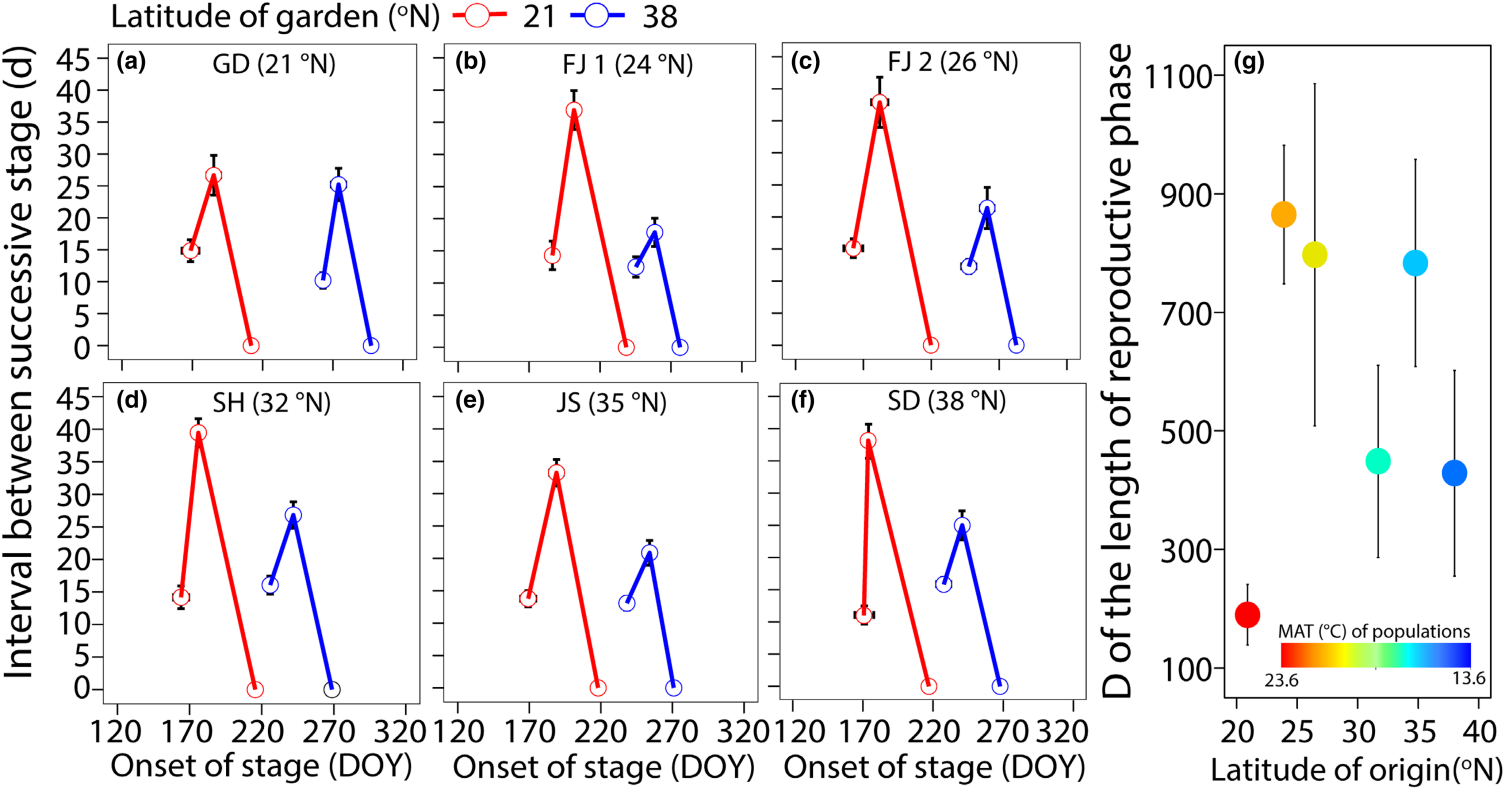
The reproductive phase of different populations, (a) Guangdong (GD, 21° N), (b) Fujian 1 (FJ 1, 24° N), (c) Fujian 2 (FJ 2, 26° N), (d) Shanghai (SH, 32° N), (e) Jiangsu (JS, 35° N), and (f) Shandong (SD, 38° N), in the low latitude (red lines) and the high latitude (blue lines) garden. Each point presents the onset (mean ± SE) of individual phenological stages (flag leaf, flowering, and mature from left to right) and the interval between successive stages (mean ± SE). (g) The scope of plastic response (D) of the length of reproductive phase among different populations. The color of the points presents the mean annual temperature (MAT) of populations.

We quantified the response of the reproductive phase in *S. alterniflora* to varying environmental conditions as the onset of each phenological stage and interval between successive stages (Post et al., 2008). We calculated the onset time of each stage as the number of days from January 1 and the start of a give stage (Schwartz, 2013). We calculated the interval between stages as the difference between the onset time of each stage and the next stage, specifically from flag leaf to flowering and from flowering to maturity. We correlated the interval between stages with onset time to qualify the length of reproductive phase of each population. The area of the polygon formed by the extension lines of each stage's representative point intersecting perpendicularly with the x‐axis was used to quantify the length of reproductive phase, with a larger area indicating a longer reproductive phase (as shown in Figure 2). This method of defining plant life history as two‐dimensional polygons allows for a direct and intuitive representation of the length and response in life history, even if the quantified results (such as the length of reproductive phase) may exceed the actual observation period of the experiment (Post et al., 2008). From this, we calculated the scope of plastic response (D) of the length of reproductive phase and the Phenotypic Plasticity Index (PI_V_) of onset time of each stage for each seed family from each population, where D = maximum value between gardens – minimum value between gardens (Stearns, 1992); PI_V_ = (maximum value between gardens – minimum value between gardens)/maximum value between gardens. The PIv ranges from 0 to 1, with 0 indicating no plasticity and 1 indicating the highest plasticity (Ren et al., 2020; Valladares et al., 2007).

We compared the mean of the D of the length of reproductive phase among populations; additionally, we conducted linear mixed effect model on the D of the length of reproductive phase, including population as the fixed effect and seed family as the random effect. To determine the phenological stage that contributes most to variation in reproductive phase, we compared the mean of the PI_V_ of the onset time of each stage per population, separately. Next, we conducted linear mixed effect model on the PI_V_ of the onset time, including stage as the fixed effect, and the seed family nested within population as the random effect.

We compared the mean of the inflorescence biomass between gardens per population, separately. Next, we conducted linear mixed effect model on the inflorescence biomass, including garden site, population and their interaction as the fixed effects, and the seed family nested within population as the random effect. We estimated the correlation between the plasticity of reproductive phase and fitness with Pearson correlation coefficient via using population means in each garden.

All above linear mixed effect models were created with the lmer function in the lme4 package (Bates et al., 2015). Type III ANOVA with Satterthwaite's method was used in the test, and we assessed normality using the Shapiro–Wilk's *tests* with the shapiro.test function in the stats package (Royston, 1995). We performed all analyses with R statistical software (R Development Core Team, 2018).

## 3 RESULTS

**FIGURE 3:**
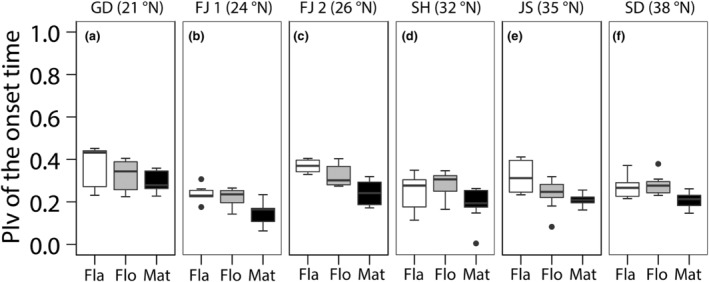
The Phenotypic Plasticity Index (PIv) of the onset time among phenological stages for each population. (a) Guangdong (GD, 21° N), (b) Fujian 1 (FJ 1, 24° N), (c) Fujian 2 (FJ 2, 26° N), (d) Shanghai (SH, 32° N), (e) Jiangsu (JS, 35° N), and (f) Shandong (SD, 38° N).

**FIGURE 4:**
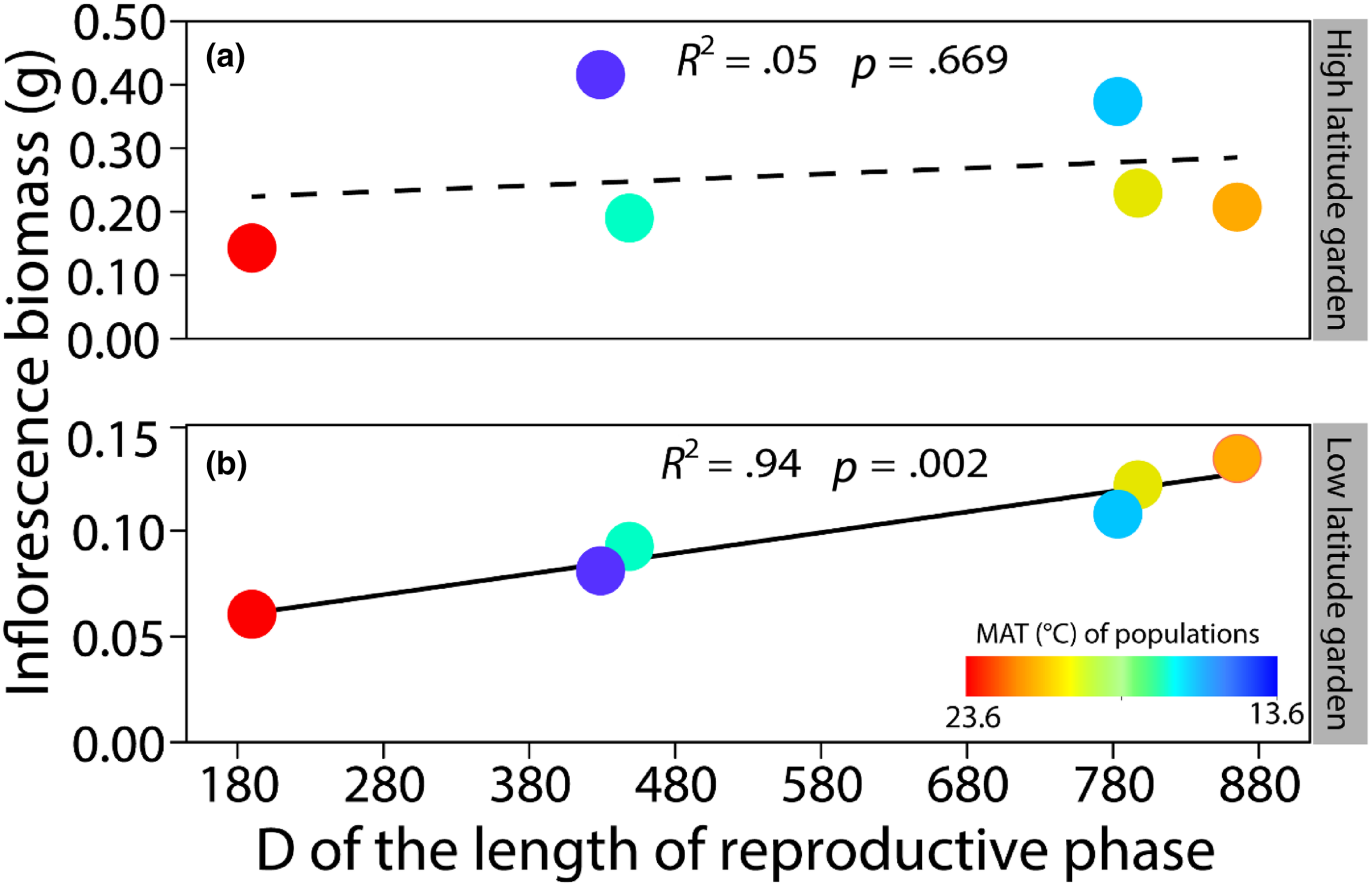
The correlation between the scope of plastic response (D) of the length of the reproductive phase against the inflorescence biomass in the high latitude garden (a) and low latitude one (b). Each point represents an individual population. The color of the points indicates the mean annual temperature (MAT) of populations. Solid line indicates significant correlation, and dash line indicates non‐significant correlation.

We found that the onset time of the three phenological stages was earlier and the length of reproductive phase was longer in the low‐latitude garden than in the high‐latitude garden for all six populations (Figure 2a–f). The scope of plastic response (D) of the length of reproductive phase was significantly different among populations (Table 1a; *F*_5,24.8_ = 2.64, *p* = .048), and lowest in the southernmost population compared to the others (Figure 2g). The Phenotypic Plasticity Index (PI_V_) of the onset time was significantly different among stages (Table 1b; *F*_2,80.9_ = 69.25, *p* < .001), and the onset time of maturity was lower than the onset time for the emergence of the flag leaf and of flowering for all six populations (Figure 3a–f). Furthermore, the D of the length of the reproductive phase was positively related to the inflorescence biomass in the low‐latitude garden across populations but not in the high latitude one (Figure 4; low latitude garden: *F*_1,4_ = 60.10, *p* = .002; high latitude garden: *F*_1,4_ = 0.21, *p* = .669). The southernmost (marginal) population from tropic had the lowest plasticity of the length of reproductive phase with the lowest inflorescence biomass than other ones (Figure 4b).

**TABLE 1:** Summary statistics of linear mixed effect models for (a) scope of plastic response (D) of the length of reproductive phase, population as the fixed effect, seed family as the random effect; (b) Phenotypic Plasticity Index (PI_V_) of the onset time, life‐history stage as the fixed effect, the seed family nested within population as the random effect; (c) inflorescence biomass, garden site, population and their interaction as the fixed effects, the seed family nested within population as the random effect.

Source	df	*F*	*p* Value
Population (fixed)	5, 24.8	2.64	**.048**
Seed family (random)	1		.165
Stage (fixed)	2, 80.9	69.25	**<.001**
Population (random)	1		**.008**
Population/seed family (random)	1		**<.001**
Garden (fixed)	1, 53.0	68.84	**<.001**
Population (fixed)	5, 66.2	1.00	.427
Garden * population (fixed)	5, 52.4	5.92	**<.001**
Population (random)	1		1.000
Population/seed family (random)	1		.653

*Note*: Significant *p* values (*p* < .05) are in bold.

## 4 DISCUSSION

Since its introduction into China, the widespread invasive plant, *S. alterniflora*, has rapidly expanded its range along a broad latitude (and thus climate) gradient (Strong & Ayres, 2013). Our use of multiple populations across this gradient, together with common gardens at the extremes of this distribution, allows us to quantify variation in the reproductive phase of *S. alterniflora*. We focused on the variation in the reproductive phase, which comprises three successive stages. These stages affect the resource trade‐off between vegetation and reproduction, with important ecological consequences for the population fitness of *S. alterniflora* between common gardens. Overall, we found onset of the reproductive phase was advanced and its length prolonged in the low latitude garden compared to the high latitude one. And the time of mature could be key in resulting in this variation between gardens. Marginal population from tropic had the lowest plasticity in reproductive phase than other ones, and such plastic responses were positively correlated with fitness.

Many long‐term studies showed that the timing of life‐history stages in different ecosystems has changed significantly and was likely related to climate change, especially the rising temperature (Parmesan & Yohe, 2003; Walther et al., 2002). We found that seedlings from all six populations reach the reproductive phase sooner and had a more prolonged reproductive phase in the warmer lower latitude common garden than the cooler higher latitude garden. This result is consistent with studies that indicated that the timing of life‐history events was advanced under warming conditions, likely because higher temperatures enhanced the physiological metabolism of plants (Anderson et al., 2012; Menzel et al., 2006; O'Connell et al., 2019). In addition, higher temperatures may prolong the length of life‐history events, as they typically correlate with extended growing seasons (Pau et al., 2011). However, high temperature may also reduce the sexual reproduction of *Spartina* species by terminating caryopsis development (Gallego‐Tévar et al., 2019; Infante‐Izquierdo et al., 2021; León‐Osper et al., 2020).

When we compared the plasticity of the length of reproductive phase among geographical populations of *S. alterniflora*, we found they generally shifted in the same direction toward the warmer low latitude garden. However, the marginal population from tropic had the lowest plasticity. Such differences among populations likely reflect genetic divergence and suggest that local environments may have selected for different capacity of shift in life history. Our result is inconsistent with the climatic variability hypothesis (CVH), which reported that populations from high latitudes and those experience greater fluctuating environmental conditions should have higher phenotypic plasticity (Hendry, 2016; Molina‐Montenegro & Naya, 2012). Nevertheless, lower levels of plasticity have been proposed at the edges of species' ranges compared to the central regions, primarily attributed to factors such as limited additive genetic variation, smaller population sizes, genetic drift, or founder effects (Mägi et al., 2011). In our study, the southernmost (marginal) population has experienced longer growing seasons and lower thermal variation (Appendix S1: Table S1); therefore, plants may have low plasticity in life history (Pau et al., 2011). As for the northernmost population, whose low level of plasticity in life history may be resulted from the physiological adaptations to freezing temperatures (Appendix S1: Table S1) (Cooper et al., 2019).

Different life‐history stages may exhibit differing levels of plasticity in response to environment (Augspurger & Zaya, 2020). Our results reveal that there was less plasticity in the timing of maturity compared to the other life stages in all the six populations. The timing of maturity can directly affect offspring production, and plasticity may be lower for traits directly related to fitness (Castillo et al., 2014; Ren et al., 2020). Additionally, for the temperate plants, fall phenology is less sensitive to temperature variation than spring phenology (such as flowering) (Cooper et al., 2019). And advances in early stages may move subsequent ones into cooler temperature, reducing temperature difference between gardens (Haggerty & Galloway, 2011). The lower plasticity in the timing of maturity led to a larger interval between the flowering time and time to maturity. As a result, we can conclude that the timing of maturity may be the key phenological stage by which reproduction is altered by *S. alterniflora* when faced with changing temperatures. Previous studies have demonstrated that different stages, such as leaf and flowering phenology, could be key in the life‐history variation (Li et al., 2016; Post et al., 2008). However, our results indicated that it may be more reasonable to emphasize the crucial role of mature. When we compared the plasticity in the timing of maturity among populations, we found the advancement of the timing of maturity of five populations from the temperate and subtropical zones was less than the marginal one from tropic. This could be because the populations from the temperate or subtropical zones are subject possible frost damage, whereby natural selection might already favor earlier maturity (Pau et al., 2011).

In our study, all the six populations advanced and prolonged their reproductive phase in the higher temperature environment, but this did not influence our measure of fitness, which was lower in the low latitude garden (Table 1c, Appendix S1: Figure S1). Therefore, with the reduced inflorescence biomass in all the six populations, the variation in the reproductive phase of *S. alterniflora* in our study could be non‐adaptive (Dudley & Schmitt, 1996). Besides, we found a significantly positive correlation between plasticity of the length of reproductive phase with inflorescence biomass across populations; this correlation only appeared in the low latitude garden after experiencing warming, while in both gardens, the marginal population from tropic with the lowest plasticity had the lowest fitness. Low level plasticity of this population may constrain its capacity to cope successfully with changing environment, resulting in the reduction of fitness. Our result thus provided a novel view to understand the center–periphery hypothesis by integration across a plant's life history, that marginal populations at range edge exhibit lower fitness and may be subject to reductions (Angert, 2006; Villellas et al., 2013). Thus, in the scenario of continuum warming in the future, the rapid geographic spread of invasive *S. alterniflora* in China may be slow down. Meanwhile, such non‐adaptive plasticity to warming suggests that evolutionary change would be required for *S. alterniflora* reproductive phase to enhance fitness under the projected warmer conditions.

Invasive *S. alterniflora* is a foundation and cosmopolitan species in China coastal wetland, whose variation in life history would have crucial implication for the plant fitness, species interaction, and the ecosystem functions. For the plant fitness, our result has suggested the correlation between plasticity of reproductive phase with fitness trait across *S. alterniflora* populations. Understanding the variation in the reproductive phase can provide insights into how species allocate resources to different life‐history stages, and it may help to predict the vulnerability of species under warming (Guo et al., 2023). For the species interaction, the advanced flag leaf under warming may not only increase the risk of frost damage to leaf tissue but also contribute to greater fitness in the competition with native species (Wang et al., 2014; Zhang et al., 2015). Variation in the timing of flowering and mature may have critical consequences, such as the plant–pollinator/herbivore relationships, seed dispersal, and species interactions because of the temporal mismatches (Guo et al., 2023). For the ecosystem functions, longer growing season would result in greater carbon sequestration (Li et al., 2016), implicating for the coastal wetland restoration and management. Variation in flag leaf, particularly in flowering and mature timing, may alter the ecological relationships among plants, pollinators, and herbivores, consequently reshaping communities and ecosystems in the future with rising temperature. Our study presents an integrated view of reproductive phase comprising three successive stages to improve our understanding and prediction of the distribution dynamic of invasive foundation species and its ripple effects on community and ecosystem under continuum warming. A possible caveat to our study is that we had only two common gardens, whereas more intermediate sites would have allowed us a clearer picture of the range of variation in response to warming.

## 5 CONCLUSION

We find that *S. alterniflora* can plastically alter its life history in response to changing environmental conductions. The uneven response in the three successive stages resulted in variation of reproductive phase, while lower plasticity in the timing of maturity made it a key stage underlying life‐history variation. The lowest life‐history plasticity and fitness of marginal population from tropic suggest that it may be less responsive to warming. This life‐history variation has genetic underpinnings, suggesting natural selection from local environments. Given that *S. alterniflora* invaded into China in 1979, the reproductive phase may be evolved after more than 40 years. As reported in previous studies that reproductive traits and their covariances of *S. alterniflora* have evolved latitudinal clines since its introduction to China, indicating adaptive responses to varying environmental conditions (Chen et al., 2023; Liu et al., 2017, 2020). Thus, the variation in reproductive phase may have long‐term and critical ecological consequences with implications for coastal wetland ecosystem functions in China.

## AUTHOR CONTRIBUTIONS

**Xincong Chen:** Conceptualization (lead); data curation (lead); funding acquisition (lead); investigation (lead); methodology (lead); visualization (lead); writing – original draft (lead); writing – review and editing (lead). **Jiayu Wang:** Conceptualization (equal); investigation (equal); methodology (equal); visualization (equal); writing – original draft (equal); writing – review and editing (equal). **Wenwen Liu:** Conceptualization (supporting); investigation (supporting); writing – original draft (supporting); writing – review and editing (supporting). **Yihui Zhang:** Conceptualization (lead); funding acquisition (equal); investigation (equal); methodology (equal); project administration (lead); resources (lead); supervision (lead); writing – original draft (equal); writing – review and editing (lead).

## FUNDING INFORMATION

This research was supported by the National Natural Science Foundation of China (Grant Nos. 32301321 to XCC, 32025026 to YHZ, 31971500 to YHZ), China Postdoctoral Science Foundation (Grant No. 2022M722653 to XCC), and the Fieldwork Funds for graduate students of Xiamen University (Grant No. 2022FG021 to JYW).

## CONFLICT OF INTEREST STATEMENT

All authors declare that they have no conflict of interest.

## Supporting information


Appendix S1

Data S1

## Data Availability

The data supporting the results and the R scripts used to generate the analyses have been uploaded as Appendix S1 and Data S1 for review.
