# Supplementary material for: Limited life‐history plasticity in marginal population of an invasive foundation species: Unraveling the genetic underpinnings and ecological implications
Source: Ecol Evol. 2024 Jun 8;14(6):e11549. doi: 10.1002/ece3.11549 (PMC11161825; doi:10.1002/ece3.11549)
Supplement: Supplementary file 1 — Appendix S1 [file ECE3-14-e11549-s002.docx]

Supplementary Materials

Limited life history plasticity in marginal population of an invasive foundation species: unraveling the genetic underpinnings and ecological implications

Xincong Chen, Jiayu Wang, Wenwen Liu, and Yihui Zhang

**Appendix S1**

**Supplementary Table**

**Table S1.** Climatic data and locations information for the common garden (C) and sampled populations in the field (P). Mean annual temperature (MAT), mean coldest daily temperature (MCDT), mean warmest daily temperature (MWDT), and temperature annual range (TAR, interval between MCDT and MWDT) data are from long-term averages (1981-2019) for each population and from 2019 in each common garden.

| Experiment | Locations | Climate zones | Latitude | Longitude | MAT  (°C) | MCDT  (°C) | MWDT  (°C) | TAR |
| --- | --- | --- | --- | --- | --- | --- | --- | --- |
| C | Guangdong | Tropic | N21 | E110 | 28.0 | 11.8 | 38.0 | / |
| P |  |  |  |  | 23.6 | 13.4 | 31.9 | 18.5 |
| P | Fujian 1 | Subtropics | N24 | E117 | 21.5 | 11.2 | 30.5 | 19.3 |
| P | Fujian 2 | Subtropics | N26 | E120 | 19.5 | 7.7 | 32.8 | 25.1 |
| P | Shanghai | Subtropics | N32 | E122 | 16.9 | 4.6 | 31.6 | 27.0 |
| P | Jiangsu | Temperate | N35 | E119 | 14.3 | 1.4 | 32.3 | 30.9 |
| P | Shandong | Temperate | N38 | E119 | 13.6 | -3.8 | 31.2 | 35.0 |
| C |  |  |  |  | 17.1 | -3.3 | 37.3 | / |

**Supplementary Figure**

**
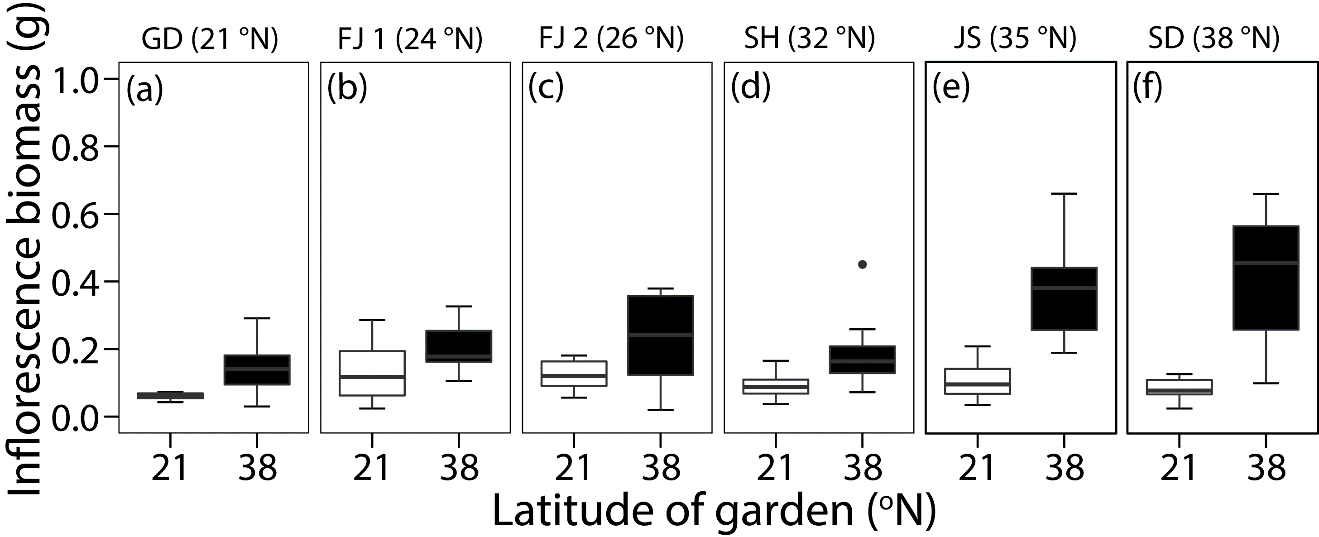
**

**Figure S1.** The inflorescence biomass between gardens for each population. (a) Guangdong (21 °N), (b) Fujian 1 (24 °N), (c) Fujian 2 (26 °N), (d) Shanghai (32 °N), (e) Jiangsu (35 °N), and (f) Shandong (38 °N).
